# Supplementary material for: Maximising environmental savings from silicon photovoltaics manufacturing to 2035
Source: Nat Commun. 2026 Feb 3;17:2311. doi: 10.1038/s41467-026-69165-x (PMC12976067; doi:10.1038/s41467-026-69165-x)
Supplement: Supplementary file 1 — Supplementary Information [file 41467_2026_69165_MOESM1_ESM.pdf]

# Maximising Environmental Savings from Silicon Photovoltaics Manufacturing to 2035

## Supplementary Information

Bethany L. Willis[1], Oliver M. Rigby[1], Sophie L. Pain[2], Nicholas E. Grant[2], John D. Murphy[3,2], Ruy S. Bonilla[4], Neil S. Beattie[1,*]

[1] School of Engineering, Physics and Mathematics, Northumbria University, Newcastle upon Tyne, NE1 8ST, United Kingdom

[2] School of Engineering, University of Warwick, Coventry, CV4 7AL, United Kingdom

[3] School of Engineering, University of Birmingham, Edgbaston, Birmingham, B15 2TT, United Kingdom

[4] Department of Materials, University of Oxford, Oxford, OX1 4NH, United Kingdom

[*]corresponding author: neil.beattie@northumbria.ac.uk

This Supplementary Information document contains data for the conduct of the results presented in the main manuscript. Life Cycle Inventories and calculations can be found in the Supplementary Data and figure data in the Source Data file. Any further data requests or questions are welcome upon request from the corresponding author: neil.beattie@northumbria.ac.uk

## Table Of Contents:


| Page | Title | Content |
|---|---|---|
| Page 2 | Supplementary Table 1 | Assumptions used for the Life Cycle Assessment (LCA) modelling. |
| Page 3 | Supplementary Table 2 | Glossary for the 16 investigate environmental impact categories used for the Environmental Footprint (EF) v3.1 impact assessment. |
| Page 4 | Supplementary Table 3 | Raw Life Cycle Assessment results for the environmental impact of manufacturing 1 $W_p$ PERC and TOPCon PV in China and transported to central Europe. |
| Page 5 | Supplementary Figure 1 | Environmental impact of manufacturing 1 $W_p$ of PERC and TOPCon modules |
| Page 6 | Supplementary Table 4 | Raw Life Cycle Assessment results comparing 1 tonne kilometre (tkm) of each transportation method considered for China to central Europe transport. |
| Page 7 | Supplementary Table 5 | Variables considered over time. |
| Page 8 | Supplementary Note 1 | Explanation of Calculation 3, Supplementary Dataset 5 |
| Page 9 | Supplementary Table 6 | Inputs used for the sensitivity to sub-grid electricity mix and *Climate change* (Fig 6) |
| Page 10 | Supplementary Table 7 | Raw results for 1 $W_p$ TOPCon manufacturing from the electricity mix composition sensitivity analysis. |
| Page 11 | Supplementary Table 8 | Inputs used for the Sensitivity to inventory changes analysis. |
| Page 12 | Supplementary Table 9 | Raw Life Cycle Assessment results for the sensitivity to the inventory analysis, (Fig 8) |
| Page 13 | Supplementary Table 10 | Monte Carlo simulation results comparing a functional unit of 1 $W_p$ PERC to TOPCon module manufacturing. |
| Page 14 | Supplementary Table 11 | Values used to scale the inventory (in $m^2$) to the functional unit for PERC and TOPCon to 2034 |
| Page 15 | Supplementary Table 12 | Projection of photovoltaic technology to 2035 and relative share of PERC and TOPCon. |
| Page 16 | Supplementary References | |

**Supplementary Table 1 | Assumptions used for the Life Cycle Assessment (LCA) modelling of Passivated Emitter Rear Cell (PERC) and Tunnel Oxide Passivated Contact (TOPCon) Photovoltaics (PV).** GLO – global, U – unit process

| Relevance | Assumptions |
|---|---|
| Life Cycle Inventory (LCI) | PERC and TOPCon wafers are manufactured in the same way (other than the additional dopant).<br>Mass of dopant in TOPCon, n-type Czochralski silicon production ($1m^2$) is 1.76E-05 kg, pedigree matrix: (3,4,5,2,1) and input "Phosphate rock, as $P_2O_5$, beneficiated, dry {GLO}\| market for phosphate rock, as P2O5, beneficiated, dry \| Cut-off, U"<br>Mass of dopant in PERC, p-type Czochralski silicon production ($1m^2$) is 4.46E-6 kg, pedigree matrix: (3,4,5,2,1) and input "Gallium, semiconductor-grade {GLO}\| market for gallium, semiconductor-grade \| Cut-off, U"<br>PERC and TOPCon wafer thicknesses are the same. (i.e. kg poly-silicon input to 1 kg Czochralski-silicon production is the same) See Supplementary Table 5<br>PERC and TOPCon cell manufacturing produces the same waste outputs<br>When modelling TOPCon Cell, the absence of "Phosphine" from the Ecoinvent database is substituted with the mass of hydrogen and phosphane.<br>PERC and TOPCon modules consist of identical module components<br>PERC and TOPCon modules have the same mass, making their transportation inputs identical<br>Unchanged inventories from Muller et al., 2021[1] are representative of the technological status in the modelled year. (e.g. metallurgical grade silicon production)<br>Lifetime is equal for PERC and TOPCon – See Supplementary Table 5<br>Degradation is equal for PERC and TOPCon – See Supplementary Table 5 |
| Functional Unit (per Watt peak, $W_p$) | The Functional unit is calculated using a formula from Muller et al., 2021[1], referred to here as Supplementary Equation 1:<br>$E_{total} = \sum_{y=2}^{T}((1-\mathrm{DR}_2)^{y-1} \times (1-\mathrm{DR}_1) \times I \times A \times \eta \times \mathrm{PR}_i) + (1-\mathrm{DR}_1) \times I \times A \times \eta \times \mathrm{PR}_i \quad (1)$<br>Where, $E_{total}$ is the total electricity output over the modules lifetime, $T$ is the module lifetime, $\mathrm{DR}_1$ is the degradation in the first year, $\mathrm{DR}_2$ is the annual degradation after year one, $I$ is the irradiation, $A$ is the module area, $\eta$ is the module efficiency, and $\mathrm{PR}_i$ is the performance ratio.<br>Insolation is chosen as 1000 kWh $m^{-2}yr^{-1}$, a conservative, low irradiation estimate for European locations.<br>Performance ratio is set as 0.8. (following guidance from the International Energy Agency (IEA) LCA in PV guidelines)[2]<br>Area of a module is $1m^2$ when calculating the functional unit.<br>Degradation and lifetime are the same for PERC and TOPCon modules |
| Manufacturing Location | Manufacturing capacity and processing yield achieved in China can be repeated in other manufacturing locations investigated.<br>Assume the average grid mix for each location is representative and appropriate for the manufacturing location scenario – a sensitivity analysis is conducted to account for differences due to this assumption.<br>Models for future electricity mixes for each location are taken from the US Energy Information Administration (EIA) International Energy Outlook (IEO), 2023, Low zero-carbon cost scenario[3]<br>Due to the absence of Europe in the EIA database the model for Western-Europe is used to represent European manufacturing |
| kWh, $CO_2$ Savings Calculation | Only the 12-year time period considered in the study is considered for this comparison<br>The "Solar Photovoltaic" electricity mix consists of PERC and TOPCon only |
| Sensitivity and Uncertainty Analysis | The value of 5 mg $W^{-1}$ silver usage for sustainable multi-terawatt PV production[4] is applicable for PERC and TOPCon modules<br>The value for electricity consumption during wafer fabrication is considered as proportional to the thickness of the wafer. This is determined using the percentage change in poly-silicon consumption per mono-silicon wafer<br>Changes made to Silver use, Wafer electricity consumption and Silane usage do not affect the efficiency of the cells |

**Supplementary Table 2 | Glossary for the 16 investigate environmental impact categories used for the Environmental Footprint (EF) v3.1 impact assessment.** References[5, 6] have been used to provide descriptions of each impact category.
eq. - equivalent

| Impact Category | Units | Description |
|---|---|---|
| Acidification | moles $H^+$ eq. | Describes the potential impact on the acidity of the air, water and soil resulting from the emission for emissions. |
| Climate change | kg carbon dioxide eq. | Describes the potential contribution towards average global temperature increases due to greenhouse gas emissions. |
| Ecotoxicity, freshwater | comparative toxic unit ecosystems | Describes the potential toxic impact of emissions on microorganisms living in freshwater ecosystems. |
| Particulate matter | disease incidence | Describes the potential impact on human health resulting from the emission of small particles (particulate matter) and its precursors to the atmosphere. |
| Eutrophication, marine | kg N eq. | Describes the potential impact of excess nutrients in a marine ecosystem and their contribution to eutrophication. (environmental degradation due to the depletion of oxygen in the ecosystem resulting from overgrowth of algae and plants stimulated by an excess of nutrients) |
| Eutrophication, freshwater | kg P eq. | Describes the potential impact of excess nutrients in a freshwater ecosystem and their contribution to eutrophication. (environmental degradation due to the depletion of oxygen in the ecosystem resulting from overgrowth of algae and plants stimulated by an excess of nutrients) |
| Eutrophication, terrestrial | moles N eq. | Describes the potential impact of excess nutrients in a terrestrial ecosystem and their contribution to eutrophication. (environmental degradation due to the depletion of oxygen in the ecosystem resulting from overgrowth of algae and plants stimulated by an excess of nutrients) |
| Human toxicity, cancer | comparative toxic unit for human toxicity | Describes the potential impact on human health caused by exposure to chemicals and substances which increase the risk of cancer. |
| Human toxicity, non-cancer | comparative toxic unit for human toxicity | Describes the potential impact on human health caused by exposure to chemicals and substances which may cause non-cancerous health effects. |
| Ionising radiation | kilobecquerels U-235 eq. | Describes the potential impact on human health due to exposure to radioactive substances. |
| Land use | Points | Describes the impact of land transformation and potential changes to soil quality. |
| Ozone depletion | kg chlorofluorocarbon-11 eq. | Describes the potential damage to the ozone layer resulting from emissions that deplete and damage the ozone layer. |
| Photochemical ozone formation | kg non-methane volatile organic compound eq. | Describes the potential impact on organisms as a result of ozone emissions in the lower atmosphere (troposphere). |
| Resource use, fossils (Fossil use) | megajoules | Describes the consumption and depletion of non-renewable, fossil fuel resources. |
| Resource use, minerals and metals (Metal use) | kg Sb eq. | Describes the consumption and depletion of non-renewable, non-fossil resources like minerals and metals. |
| Water use | $m^3$ of water deprived | Describes the impact of depleting water from natural sources. |

**Supplementary Table 3 | Raw Life Cycle Assessment (LCA) results for the environmental impact of manufacturing 1 $W_p$ PERC and TOPCon PV module in China and transported to central Europe.**
eq. - equivalent

| Impact Category | Unit | PERC | TOPCon |
|---|---|---|---|
| Acidification | moles $H^+$ eq. | 0.005374 | 0.005046 |
| Climate change | kg carbon dioxide eq. | 0.813977 | 0.761132 |
| Ecotoxicity, freshwater | comparative toxic unit ecosystems | 4.615432 | 4.327354 |
| Particulate matter | disease incidence | 6.22E-08 | 5.84E-08 |
| Eutrophication, marine | kg N eq. | 0.001029 | 0.000971 |
| Eutrophication, freshwater | kg P eq. | 0.000217 | 0.000205 |
| Eutrophication, terrestrial | moles N eq. | 0.011186 | 0.010548 |
| Human toxicity, cancer | comparative toxic unit for human toxicity | 1.61E-09 | 1.49E-09 |
| Human toxicity, non-cancer | comparative toxic unit for human toxicity | 1.07E-08 | 1.01E-08 |
| Ionising radiation | kilobecquerels U-235 eq. | 0.036659 | 0.032486 |
| Land use | points | 4.35434 | 4.116248 |
| Ozone depletion | kg chlorofluorocarbon-11 eq. | 1.6E-08 | 1.27E-08 |
| Photochemical ozone formation | kg non-methane volatile organic compound eq. | 0.00418 | 0.003951 |
| Resource use, fossils (Fossil use) | megajoules | 8.539474 | 7.958153 |
| Resource use, minerals and metals (Metal use) | kg Sb equivalent | 1.16E-05 | 1.36E-05 |
| Water use | $m^3$ of water deprived | 0.305842 | 0.250439 |

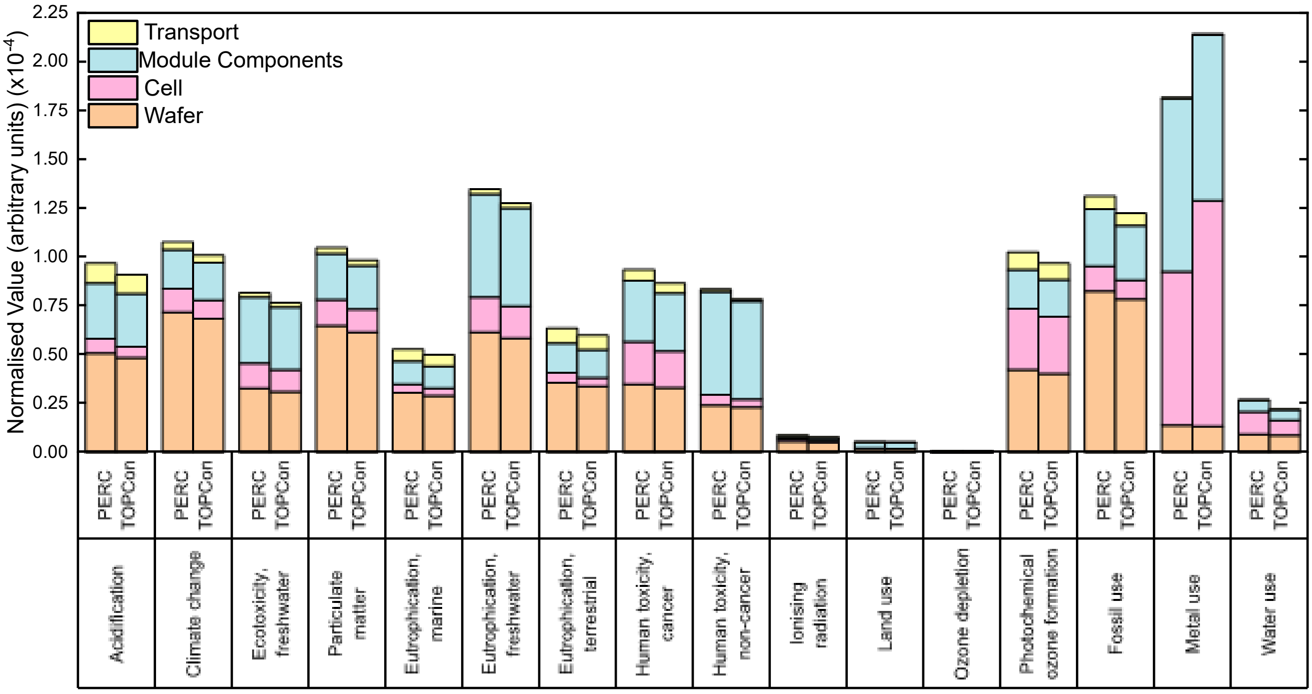


**Supplementary Figure 1 | Environmental impact of manufacturing 1 $W_p$ of PERC and TOPCon modules.** All 16 investigated environmental impact categories are considered here and presented as normalised data.

**Supplementary Table 4 | Raw results comparing 1 tonne kilometre (tkm) of each transportation method considered for China to central Europe transport.** Results are shown for the six highest value environmental impact categories shown in Fig. 2d.

| Impact Category | Unit | Transoceanic freight ship | Freight train (China) | Freight train (Europe) | Freight, lorry (China) | Freight, lorry (Europe) |
|---|---|---|---|---|---|---|
| Climate change | kg carbon dioxide eq. | 0.01172 | 0.048532 | 0.04684 | 0.19575 | 0.193682 |
| Particulate matter | disease incidence | 3.33E-10 | 1.02E-08 | 3.97E-09 | 1.55E-08 | 1.52E-08 |
| Eutrophication, freshwater | kg phosphorus eq. | 1.09E-06 | 8.33E-06 | 1.47E-05 | 1.53E-05 | 1.29E-05 |
| Photochemical ozone formation | kg non-methane volatile organic compound eq. | 0.000176 | 0.000443 | 0.000422 | 0.000907 | 0.000949 |
| Resource use, fossils (Fossil use) | megajoules | 0.147536 | 0.560263 | 0.676241 | 2.74363 | 2.71822 |
| Resource use, minerals and metals (Metal use) | kg antimony eq. | 4.35E-09 | 1.33E-07 | 1.36E-07 | 6.27E-07 | 6.19E-07 |

**Supplementary Table 5 | Variables considered over time.** These values have been obtained from the International Technology Roadmap for Photovoltaics (ITRPV), 2024[7]

| Efficiency (%) | | |
|---|---|---|
| Year | PERC | TOPCon |
| 2023 | 21.5 | 22.6 |
| 2024 | 21.6 | 22.8 |
| 2026 | 21.7 | 23.0 |
| 2028 | 21.8 | 23.4 |
| 2031 | 21.9 | 23.8 |
| 2034 | 22.0 | 24.0 |
| Silver use (kg $m^{-2}$) | | |
| Year | PERC | TOPCon |
| 2023 | 2.26E-03 | 3.62E-03 |
| 2024 | 2.11E-03 | 3.50E-03 |
| 2026 | 1.96E-03 | 3.08E-03 |
| 2028 | 1.81E-03 | 2.72E-03 |
| 2031 | 1.75E-03 | 2.54E-03 |
| 2034 | 1.57E-03 | 2.26E-03 |
| Poly-Silicon per kg Czochralski Silicon ingot production | | |
| Year | Amount (kg $m^{-2}$) | |
| 2023 | 4.89E-1 | |
| 2024 | 4.71E-1 | |
| 2026 | 4.35E-1 | |
| 2028 | 4.23E-1 | |
| 2031 | 3.92E-1 | |
| 2034 | 3.62E-1 | |
| Lifetime (years) | | |
| Year | Lifetime | |
| 2023 | 30 | |
| 2024 | 30 | |
| 2026 | 30 | |
| 2028 | 35 | |
| 2031 | 36 | |
| 2034 | 40 | |
| Degradation (%) | | |
| Year | Year 1 | Annual (+1 year) |
| 2023 | 2.0 | 0.50 |
| 2024 | 1.5 | 0.50 |
| 2026 | 1.0 | 0.50 |
| 2028 | 1.0 | 0.40 |
| 2031 | 1.0 | 0.40 |
| 2034 | 1.0 | 0.40 |

**Supplementary Note 1 |** Explanation of Calculation 3 (Supplementary Data 5)

This Note is to be used alongside Supplementary Data 5.

*Calculating the Solar Photovoltaic (PV)impact per kilowatt hour (kWh). (data used for Fig. 5)*
When calculating the Climate change impact from electricity generation, a functional unit of 1 kWh is used to calculate the impact for Passivated Emitter Rear Cell (PERC) and Tunnel Oxide Passivating Contact (TOPCon). This is found by inputting the area per kWh into the Life Cycle Assessment (LCA) software. This output is shown in Table A, which considers manufacturing in China and transported to central Europe.

Since the PV deployed between 2023-2034 is a mix of PERC and TOPCon technology, this needs to be accounted for. Table B shows how the impact per kWh is calculated considering a proportion of PERC and TOPCon values from Table A. This is done for the years 2023, 2024, 2026, 2028, 2031 and 2034. This gives the impact per kWh, an example is provided. This data is used to create Fig. 5 for Solar PV.

*Calculating the output from PERC and TOPCon PV between 2023-2035*
The area of PV deployed between 2023-2035 is considered as shown in Table C, the calculations are supported by an example.

Using Calculation 1, in Supplementary Data 3 the electricity output from 1 $m^2$ can be calculated for PERC and TOPCon by changing the parameters using Supplementary Table 5 to represent each year for both technologies. The results are shown in Table D, along with an example. When calculating the area per kWh, the area (1$m^2$) is divided by the total electricity output whereas for the area per $W_p$, the area is divided by the electricity output in year 1.

The values in Table C ($m^2$ area of PV deployed between 2023-2035) are multiplied by the values in Table D (electricity output per $m^2$) to give the values in Table E. This gives the amount of electricity produced by the deployed PV between 2023-2025. As an additional insight, Table F gives the value of electricity output over the whole PV lifetime.

*Climate change impact corresponding to kWh produced between 2023-2035*
Now that the amount of electricity output is known, the Climate change impact can be calculated. The impact per kWh of each, PERC and TOPCon, (Table A) is multiplied by the electricity output between 2023-2035 (Table E) to produce Table G. This is where the value 2.26 Gt $CO_2$ equivalent (eq.) value comes from.

As an additional insight, the impact can be found over the whole lifetime (Table H) where the value 11.00 Gt $CO_2$ eq. – notice that this is the same as the impact of deployment if all manufacturing was done in China (from Fig. 4a / Source data 5).

*Climate change impact from different locations corresponding to the kWh produced by the PV between 2023-2035*
To compare the 2.26 Gt $CO_2$ eq. value to the emissions associated with the generation of equal kWh, a similar approach is taken for each location. Table I provides the Climate change impact per kWh for each location's future electricity mix – taken from the US Energy Information Administration, international energy outlook, using a low zero-carbon cost scenario[3]. This is found by building the electricity composition for each location in each time period (Using Calculation 2 in Supplementary Data 4) in LCA software and measuring the impact per kWh.

Note: The modelling each future electricity mix is based on Germany's current electricity mix in SimaPro. Example, Wind power can be installed in the form of "wind, <1 MW turbine, onshore", "wind, >3MW turbine, onshore", "wind, 1-3MW turbine, offshore" and "wind, 1-3MW turbine, onshore", all of which will have different environmental impacts. This level of detail required for each location's individual future electricity mix is not known, only the overall electricity generation source percentage contribution to the mix (i.e. "X % of wind to make 1 kWh"). Therefore, to allow consistent modelling across locations and neglect the difference in impact due to variation within a particular electricity source's generation, the share of each electricity source is split consistent with Germany's current electricity mix, (i.e. 13.7% <1 MW onshore wind, 8.2% >3 MW onshore wind, 2.9% 1-3MW offshore wind, and 75.2% 1-3MW onshore wind). Germany was chosen as it has most of the electricity generating resources in its mix except solar which was modelled on Spain.

Multiplying the values for impact per kWh (Table I) with the number of kWh in each time period (Table E) gives the $CO_2$ eq. from producing equivalent kWh to the PV, producing the values in Table J. This is where the numbers: 62.09 Gt $CO_2$ eq. in China, 66.75 Gt $CO_2$ eq. in India, 32.69 Gt $CO_2$ eq. in the US and 27.56 Gt $CO_2$ eq. in Europe are obtained as stated in the manuscript. These values, when cared to the 2.26 Gt $CO_2$ eq. from the PV demonstrate very high $CO_2$ eq. savings.

**Supplementary Table 6 | Inputs used for the sensitivity to sub-grid electricity mix and *Climate change* results (Fig 6).** These electricity mixes are taken from the Ecoinvent v3.10 database. The results are for 1 Watt peak (Wp) manufacturing in each corresponding location and transported to central Europe.

| Location and Carbon intensity | PERC (kg $CO_2$ eq.) | TOPCon (kg $CO_2$ eq.) |
|---|---|---|
| Low carbon intensity China, CN-SWG (South-West Grid) | 0.48 | 0.45 |
| Reference China, CN - (Average) | 0.81 | 0.76 |
| High carbon intensity China, CN-NECG (North-East Grid) | 1.07 | 1.00 |
| Low carbon intensity India, IN-North-Eastern | 0.76 | 0.71 |
| Reference India, IN - (Average) | 1.02 | 0.95 |
| High carbon intensity India, - IN-Eastern | 1.10 | 1.03 |
| Low carbon intensity US, US-NPCC (Northeast Power Coordinating Council) | 0.41 | 0.38 |
| Reference US, US - (Average) | 0.55 | 0.51 |
| High carbon intensity US, US-HICC (Hawaii Island Coordinating Council) | 0.79 | 0.74 |
| Low carbon intensity EU, CH - Switzerland | 0.27 | 0.24 |
| Europe - (Average) | 0.44 | 0.40 |
| High carbon intensity EU, PL - Poland | 0.76 | 0.71 |

**Supplementary Table 7 | Raw Life Cycle Assessment (LCA) results for 1 $W_p$ TOPCon manufacturing from electricity mix composition sensitivity analysis.** Calculations are provided in Supplementary Data 6. eq. – equivalent, CTU - comparative toxic units, CFC – chlorofluorocarbon, NMVOC – non methane volatile organic compounds

| Impact Category | Unit | Equal | Coal | Hydro-power | Nuclear | Photo-voltaic | Wind | Biogas | Gas | Geo-thermal | Oil |
|---|---|---|---|---|---|---|---|---|---|---|---|
| Acidification | Moles H eq. | 2.43E-03 | 2.48E-03 | 2.41E-03 | 2.40E-03 | 2.42E-03 | 2.40E-03 | 2.43E-03 | 2.42E-03 | 2.41E-03 | 2.48E-03 |
| Climate change | kg carbon dioxide eq. | 4.27E-01 | 4.48E-01 | 4.21E-01 | 4.17E-01 | 4.19E-01 | 4.17E-01 | 4.21E-01 | 4.33E-01 | 4.18E-01 | 4.43E-01 |
| Ecotoxicity, freshwater | CTU ecosystems | 3.38E+00 | 3.42E+00 | 3.36E+00 | 3.35E+00 | 3.38E+00 | 3.36E+00 | 3.37E+00 | 3.37E+00 | 3.38E+00 | 3.37E+00 |
| Particulate matter | disease incidence | 2.43E-08 | 2.43E-08 | 2.42E-08 | 2.41E-08 | 2.42E-08 | 2.41E-08 | 2.43E-08 | 2.41E-08 | 2.42E-08 | 2.49E-08 |
| Eutrophication, marine | kg N eq. | 4.27E-04 | 4.40E-04 | 4.23E-04 | 4.21E-04 | 4.23E-04 | 4.20E-04 | 4.23E-04 | 4.26E-04 | 4.22E-04 | 4.29E-04 |
| Eutrophication, freshwater | kg P eq. | 2.30E-04 | 2.39E-04 | 2.26E-04 | 2.24E-04 | 2.25E-04 | 2.24E-04 | 2.25E-04 | 2.24E-04 | 2.25E-04 | 2.24E-04 |
| Eutrophication, terrestrial | moles N eq. | 4.61E-03 | 4.74E-03 | 4.56E-03 | 4.54E-03 | 4.57E-03 | 4.54E-03 | 4.59E-03 | 4.60E-03 | 4.56E-03 | 4.64E-03 |
| Human toxicity, cancer | CTU human toxicity | 1.47E-09 | 1.47E-09 | 1.46E-09 | 1.45E-09 | 1.47E-09 | 1.48E-09 | 1.46E-09 | 1.48E-09 | 1.49E-09 | 1.48E-09 |
| Human toxicity, non-cancer | CTU human toxicity | 8.75E-09 | 8.96E-09 | 8.71E-09 | 8.69E-09 | 8.80E-09 | 8.71E-09 | 8.71E-09 | 8.71E-09 | 8.70E-09 | 8.72E-09 |
| Ionising radiation | kilobecquerels U-235 eq. | 4.37E-02 | 4.20E-02 | 4.25E-02 | 5.94E-02 | 4.20E-02 | 4.18E-02 | 4.19E-02 | 4.18E-02 | 4.20E-02 | 4.19E-02 |
| Land use | points | 4.48E+00 | 4.44E+00 | 4.42E+00 | 4.40E+00 | 4.41E+00 | 4.41E+00 | 4.43E+00 | 4.41E+00 | 4.41E+00 | 4.42E+00 |
| Ozone depletion | kg CFC11 eq. | 1.05E-08 | 1.04E-08 | 1.05E-08 | 1.04E-08 | 1.06E-08 | 1.04E-08 | 1.04E-08 | 1.10E-08 | 1.04E-08 | 1.09E-08 |
| Photochemical ozone formation | kg NMVOC eq. | 2.48E-03 | 2.50E-03 | 2.46E-03 | 2.45E-03 | 2.46E-03 | 2.46E-03 | 2.47E-03 | 2.49E-03 | 2.46E-03 | 2.52E-03 |
| Resource use, fossils (Fossil use) | megajoules | 5.61E+00 | 5.78E+00 | 5.52E+00 | 5.81E+00 | 5.49E+00 | 5.46E+00 | 5.48E+00 | 5.72E+00 | 5.48E+00 | 5.77E+00 |
| Resource use, minerals and metals (Metal use) | kg Sb eq. | 1.39E-05 | 1.39E-05 | 1.39E-05 | 1.39E-05 | 1.41E-05 | 1.39E-05 | 1.39E-05 | 1.39E-05 | 1.39E-05 | 1.39E-05 |
| Water use | $m^3$ water deprived | 2.57E-01 | 2.68E-01 | 2.54E-01 | 2.57E-01 | 2.55E-01 | 2.53E-01 | 2.53E-01 | 2.54E-01 | 2.53E-01 | 2.64E-01 |

**Supplementary Table 8 | Inputs used for the sensitivity to inventory changes analysis for PERC and TOPCon PV.** These values and their relative benchmark correspond to the data show in Fig. 8. kWh – kilowatt hours

| Feature | Reference | Relative Change (%) (PERC) | Original Value (PERC) | New Value (PERC) | Relative Change (%) (TOPCon) | Original Value (TOPCon) | New Value (TOPCon) |
|---|---|---|---|---|---|---|---|
| Efficiency Increase | ITRPV 2024,[7] | + 12.6 (%) | 21.50 (%) | 24.20 (%) | + 15.9 (%) | 22.60 (%) | 26.20 (%) |
| Silver Reduction | Hagel et al., 2021,[4] | - 66.5 (%) | 2.26E-03 (kg $m^{-2}$) | 7.57E-04 (kg $m^{-2}$) | - 78.0 (%) | 3.62E-03 (kg $m^{-2}$) | 7.96E-04 (kg $m^{-2}$) |
| Wafer Electricity Reduction | ITRPV 2024,[7] | - 26.0 (%) | 123.75 (kWh) | 91.58 (kWh) | - 26.0 (%) | 123.75 (kWh) | 91.58 (kWh) |
| Silane Reduction | Yoon et al., 2024[8] | - 14.4 (%) | 2.83E-03 (kg) | 2.42E-03 (kg) | - 14.4 (%) | 3.11E-03 (kg) | 2.66E-03 (kg) |

| Feature | Change and relative benchmark |
|---|---|
| Efficiency Increase | Change from stabilised module efficiency in 2023 to stabilised cell efficiency in 2034, both according to International Technology Roadmap for Photovoltaics (*ITRPV), 2024*[7]. The percentage conversion efficiency of PERC increases to 24.20% whilst TOPCon improves to 26.20%. |
| Silver Reduction | Change from ITRPV, 2024[7] value to 5 milligrams per watt based on *Hagel et al., 2021*,[4] who made that comment that this value is a target for achieving sustainable multi-terawatt PV production. |
| Wafer Electricity Reduction | Change from *Muller et al, 2024*[1] values by 26% guided by the *ITRPV, 2024* roadmap |
| Silane Reduction | Change from *Muller et al, 2024*[1] value (PERC) and TOPCon manufacturer data (TOPCon) by 14.4% which can be considered a conservative value based on the findings by *Yoon et al., 2024*[8]. |

**Supplementary Table 9 | Raw Life Cycle Assessment (LCA) results for the sensitivity to the inventory analysis, (Fig 8).** eq. – equivalent, CTU - comparative toxic units, CFC – chlorofluorocarbon, NMVOC – non methane volatile organic compounds

| | | Passivated Emitter Rear Cell (PERC) | | | | | Tunnel Oxide Passivated Contact (TOPCon) | | | | |
|---|---|---|---|---|---|---|---|---|---|---|---|
| Impact Category | Unit | Original | Efficiency | Silver use | Wafer elec. | Silane | Original | Efficiency | Silver use | Wafer elec. | Silane |
| Acidification | mol H+ eq. | 5.37E-03 | 4.78E-03 | 5.35E-03 | 4.71E-03 | 5.37E-03 | 5.05E-03 | 4.36E-03 | 5.01E-03 | 4.41E-03 | 5.04E-03 |
| Climate change | kg $CO^2$ eq. | 8.14E-01 | 7.23E-01 | 8.12E-01 | 6.88E-01 | 8.12E-01 | 7.61E-01 | 6.57E-01 | 7.58E-01 | 6.41E-01 | 7.59E-01 |
| Ecotoxicity, freshwater | CTUe | 4.62E+00 | 4.10E+00 | 4.47E+00 | 4.26E+00 | 4.61E+00 | 4.33E+00 | 3.74E+00 | 4.07E+00 | 3.98E+00 | 4.32E+00 |
| Particulate matter | disease incidence | 6.22E-08 | 5.53E-08 | 6.21E-08 | 5.29E-08 | 6.21E-08 | 5.84E-08 | 5.05E-08 | 5.82E-08 | 4.96E-08 | 5.84E-08 |
| Eutrophication, marine | kg N eq. | 1.03E-03 | 9.15E-04 | 1.02E-03 | 8.88E-04 | 1.03E-03 | 9.71E-04 | 8.39E-04 | 9.60E-04 | 8.37E-04 | 9.70E-04 |
| Eutrophication, freshwater | kg P eq. | 2.17E-04 | 1.93E-04 | 2.12E-04 | 1.94E-04 | 2.16E-04 | 2.05E-04 | 1.77E-04 | 1.96E-04 | 1.83E-04 | 2.04E-04 |
| Eutrophication, terrestrial | moles N eq. | 1.12E-02 | 9.94E-03 | 1.11E-02 | 9.68E-03 | 1.12E-02 | 1.05E-02 | 9.11E-03 | 1.04E-02 | 9.12E-03 | 1.05E-02 |
| Human toxicity, cancer | CTUh | 1.61E-09 | 1.43E-09 | 1.61E-09 | 1.51E-09 | 1.60E-09 | 1.49E-09 | 1.29E-09 | 1.48E-09 | 1.40E-09 | 1.48E-09 |
| Human toxicity, non-cancer | CTUh | 1.07E-08 | 9.54E-09 | 1.07E-08 | 1.01E-08 | 1.07E-08 | 1.01E-08 | 8.71E-09 | 9.98E-09 | 9.46E-09 | 1.01E-08 |
| Ionising radiation | Kilobecquerel U-235 eq. | 3.67E-02 | 3.26E-02 | 3.65E-02 | 3.12E-02 | 3.64E-02 | 3.25E-02 | 2.81E-02 | 3.21E-02 | 2.73E-02 | 3.23E-02 |
| Land use | Points | 4.35E+00 | 3.87E+00 | 4.33E+00 | 4.12E+00 | 4.35E+00 | 4.12E+00 | 3.55E+00 | 4.08E+00 | 3.89E+00 | 4.11E+00 |
| Ozone depletion | kg CFC11 eq. | 1.60E-08 | 1.42E-08 | 1.60E-08 | 1.57E-08 | 1.60E-08 | 1.27E-08 | 1.10E-08 | 1.27E-08 | 1.25E-08 | 1.27E-08 |
| Photochemical ozone formation | kg NMVOC eq. | 4.18E-03 | 3.72E-03 | 4.16E-03 | 3.78E-03 | 4.18E-03 | 3.95E-03 | 3.41E-03 | 3.92E-03 | 3.57E-03 | 3.95E-03 |
| Resource use, fossils (Fossil use) | megajoules | 8.54E+00 | 7.59E+00 | 8.51E+00 | 7.32E+00 | 8.52E+00 | 7.96E+00 | 6.87E+00 | 7.91E+00 | 6.80E+00 | 7.93E+00 |
| Resource use, minerals and metals (Metal use) | kg Sb eq. | 1.16E-05 | 1.03E-05 | 8.41E-06 | 1.15E-05 | 1.16E-05 | 1.36E-05 | 1.18E-05 | 8.00E-06 | 1.36E-05 | 1.36E-05 |
| Water use | $m^3$ water deprived | 3.06E-01 | 2.72E-01 | 3.05E-01 | 2.92E-01 | 2.89E-01 | 2.50E-01 | 2.16E-01 | 2.50E-01 | 2.37E-01 | 2.33E-01 |

**Supplementary Table 10 | Monte Carlo simulation results comparing a functional unit of 1 Watt peak ($W_p$) PERC to TOPCon module manufacturing.** These results are calculated using a confidence level of 95% over 10,000 runs. This is based on the 2023 China (average) manufacturing scenario and transported to central Europe. Where "*A*" is the impact of 1 $W_p$ PERC manufacturing and "*B*" is the impact of 1 $W_p$ TOPCon manufacturing. SD – Standard deviation, CV – Coefficient of Variation, SEM – Standard Error of the Mean.

| Damage category | A >= B | Mean | Median | SD | CV | 2.5% | 97.5% | SEM |
|---|---|---|---|---|---|---|---|---|
| Acidification | 73.77 | 3.32E-04 | 3.29E-04 | 5.30E-04 | 1.60E+02 | -6.99E-04 | 1.39E-03 | 5.30E-06 |
| Climate change | 71.49 | 5.35E-02 | 5.25E-02 | 9.63E-02 | 1.80E+02 | -1.33E-01 | 2.47E-01 | 9.63E-04 |
| Ecotoxicity, freshwater | 76.5 | 2.91E-01 | 2.92E-01 | 4.17E-01 | 1.43E+02 | -5.30E-01 | 1.11E+00 | 4.17E-03 |
| Eutrophication, freshwater | 70.31 | 1.21E-05 | 1.20E-05 | 2.34E-05 | 1.94E+02 | -3.42E-05 | 5.83E-05 | 2.34E-07 |
| Eutrophication, marine | 71.36 | 5.86E-05 | 5.83E-05 | 1.04E-04 | 1.78E+02 | -1.45E-04 | 2.67E-04 | 1.04E-06 |
| Eutrophication, terrestrial | 71.82 | 6.46E-04 | 6.41E-04 | 1.12E-03 | 1.73E+02 | -1.56E-03 | 2.88E-03 | 1.12E-05 |
| Human toxicity, cancer | 84.99 | 1.19E-10 | 1.17E-10 | 1.18E-10 | 9.91E+01 | -1.12E-10 | 3.56E-10 | 1.18E-12 |
| Human toxicity, non-cancer | 65.73 | 6.51E-10 | 6.49E-10 | 1.68E-09 | 2.58E+02 | -2.67E-09 | 3.99E-09 | 1.68E-11 |
| Ionising radiation | 86.62 | 4.19E-03 | 4.15E-03 | 3.83E-03 | 9.16E+01 | -3.25E-03 | 1.18E-02 | 3.83E-05 |
| Land use | 69.1 | 2.42E-01 | 2.35E-01 | 5.05E-01 | 2.09E+02 | -7.58E-01 | 1.22E+00 | 5.05E-03 |
| Ozone depletion | 98.67 | 3.29E-09 | 3.23E-09 | 1.54E-09 | 4.68E+01 | 4.09E-10 | 6.48E-09 | 1.54E-11 |
| Particulate matter | 72.13 | 3.79E-09 | 3.81E-09 | 6.62E-09 | 1.74E+02 | -9.21E-09 | 1.71E-08 | 6.62E-11 |
| Photochemical ozone formation | 69.85 | 2.26E-04 | 2.23E-04 | 4.44E-04 | 1.96E+02 | -6.51E-04 | 1.12E-03 | 4.44E-06 |
| Resource use, fossils (Fossil use) | 73.69 | 5.87E-01 | 5.77E-01 | 9.62E-01 | 1.64E+02 | -1.27E+00 | 2.51E+00 | 9.62E-03 |
| Resource use, minerals and metals (Metal use) | 4.24 | -2.06E-06 | -2.07E-06 | 1.19E-06 | -5.79E+01 | -4.41E-06 | 2.70E-07 | 1.19E-08 |
| Water use | 59.25 | 5.66E-02 | 5.65E-02 | 2.52E-01 | 4.46E+02 | -4.42E-01 | 5.51E-01 | 2.52E-03 |

**Supplementary Table 11 | Values used to scale the inventory (in $m^2$) to the functional unit for PERC and TOPCon to 2034.** The Watt peak ($W_p$) functional unit is used throughout the study whereas the kilowatt hour (kWh) functional unit is used for the section heading "*Carbon Dioxide Emissions, per kWh*". The functional unit is calculated using Equation 1 (shown in Supplementary Table 1), an example of the calculation of the functional unit is provided in Supplementary Data 3.

| Year | PERC ($m^2\ W_p^{-1}$) | TOPCon ($m^2\ W_p^{-1}$) | PERC ($m^2\ kWh^{-1}$) | TOPCon ($m^2\ kWh^{-1}$) |
|---|---|---|---|---|
| 2023 | 5.93E-03 | 5.64E-03 | 2.12E-04 | 2.02E-04 |
| 2024 | 5.88E-03 | 5.57E-03 | 2.10E-04 | 1.99E-04 |
| 2026 | 5.82E-03 | 5.49E-03 | 2.08E-04 | 1.97E-04 |
| 2028 | 5.79E-03 | 5.40E-03 | 1.77E-04 | 1.65E-04 |
| 2031 | 5.77E-03 | 5.31E-03 | 1.72E-04 | 1.58E-04 |
| 2034 | 5.77E-03 | 5.26E-03 | 1.72E-04 | 1.42E-04 |

**Supplementary Table 12 | Projection of PV technology to 2035 and relative share of PERC and TOPCon.** The assumption is made that the market consists of only PERC and TOPCon in this time period. Values are based on the ITRPV 2024[7]. This is relevant for calculating the data required for Fig. 4a and Fig. 4b. $TW_p$ – Terawatt peak

| Time period | Total PV deployed ($TW_p$) | Share of PERC (%) | Share of TOPCon (%) |
|---|---|---|---|
| 2023 à 2024 | 0.7 | 70 | 30 |
| 2024 à 2026 | 1.9 | 45 | 55 |
| 2026 à 2028 | 2.6 | 23 | 77 |
| 2028 à 2031 | 4.2 | 15 | 85 |
| 2031 à 2034 | 4.8 | 3 | 97 |
| 2034 à 2035 | 1.5 | 0 | 100 |

**Supplementary References:**


[1] Müller. A., Friedrich. L., Reichel.C., Herceg. S., Mittag. M. and Neuhaus. D.H. A comparative life cycle assessment of silicon PV modules: Impact of module design, manufacturing location and inventory *Sol. Energy Mater. Sol. Cells.* **230,** 111277 (2021).

[2] Fthenakis. V. *et al.*, Methodology guidelines on life cycle assessment of photovoltaic electricity *IEA PVPS Task,* vol. **12,** (2011).

[3] Energy Information Administration (EIA) International Energy Outlook, 2023. https://www.eia.gov/outlooks/ieo/pdf/IEO2023_Narrative.pdf (2024).

[4] Haegel. N.M. *et al.*, Photovoltaics at multi-terawatt scale: waiting is not an option. *Science*. **380,** 39-42. (2023).

[5] European Union. Life Cycle Assessment & the EF methods https://green-business.ec.europa.eu/environmental-footprint-methods/life-cycle-assessment-ef-methods_en. (2024).

[6] Greenhouse Sustainability. "What are the environmental impact categories?" Greenhouse Sustainability. https://greenhouse-sustainability.com/en/articles/environmental-impact-categories/ (2024).

[7] VerbandDeutscherMaschinenUndAnlagenbau(VDMA). International Technology Roadmap for Photovoltaics (ITRPV); 15th Edition, 2024 https://www.vdma.org/international-technology-roadmap-photovoltaic (2024).

[8] Yoon. M.Y., Yeom. H.-J., Jeong. J.-R., Lee. H.-C. and Kim. J.-H. Effect of SiH4 fragments and H radicals on nc-Si: H film deposition in an inductively coupled plasma PECVD. *Surf. IInterfaces*. **60,** 106040 (2025).
