# Supplementary material for: Maximising environmental savings from silicon photovoltaics manufacturing to 2035
Source: Nat Commun. 2026 Feb 3;17:2311. doi: 10.1038/s41467-026-69165-x (PMC12976067; doi:10.1038/s41467-026-69165-x)
Supplement: Supplementary file 2 — Descriptions of Additional Supplementary Files [file 41467_2026_69165_MOESM2_ESM.pdf]

Descriptions of Additional Supplementary Files

Supplementary Data 1 : **Inventory 1 -** Life Cycle Inventory table for 1m2 tunnel oxide passivated contact (TOPCon) cell manufacturing.

Supplementary Data 2 : **Inventory 2 -** Life Cycle Inventory table for 1m2 passivated emitter rear cell (PERC) cell manufacturing.

Supplementary Data 3: **Calculation 1 -** Functional Unit Calculation using Equation 1 (Supplementary Table 1).

Supplementary Data 4: **Calculation 2 -** Calculations used to model electricity mix locations based on the US Energy Information Administration (EIA) Low zerocarbon cost scenario) electricity mix.

Supplementary Data 5 : **Calculation 3 -** Calculations used to compare Climate Change impact per kilowatt hour (kWh).

Supplementary Data 6: **Calculation 4 -** Calculations used to model the electricity mix composition for the sensitivity analysis in Fig. 7.
